# Implementation of public health policies for healthy lifestyles promotion: what Brazil should tell us?

**DOI:** 10.15171/hpp.2018.33

**Published:** 2018-07-07

**Authors:** Carlos K. B. Ferrari

**Affiliations:** Instituto de Ciências Biológicas e da Saúde (ICBS), Campus Universitário do Araguaia, Universidade Federal de Mato Grosso. Av. Valdon Varjão, 6390, Setor Industrial, Barra do Garças, 78.600-000, MT, Brazil

**Keywords:** Promotion, Developing nations, Physical activity, Diet, Pitfalls

## Abstract

The objective of this work was to update Brazilian experiences on implementation research (IR)regarding promotion of healthy lifestyles to decrease the risk of chronic non-communicable diseases (CNCD). Some Brazilian governmental activities for promotion of healthy lifestyles have been adopted around the world such as the case of the "World Physical Activity Day" and the"Walking for health program". Following the example of many other developing and developed countries, Brazilian government has been sponsored leisure-time physical activity and healthy eating programs which still were not capable of promoting massive participation, especially from workers, elderly and people from the less privileged socioeconomic classes. Although successful exercise promotion strategies have been done in Brazil, the implementation science on promotion of healthy lifestyles is still rising and more research is necessary to identify the settings, circumstances, and processes for effective and sustainable adoption of healthy dietary and exercise practices. Understanding problems, concerns and barriers for implementation of health promotion (HP) policies in Brazil could be useful for many other developing nations.

## Introduction

Dissemination and implementation are complex social and organizational processes by which new scientific advances can be translated and disseminated to people, settings, and communities affecting and improving public health.^1^ Implementation research (IR) should be stimulated in research institutions, schools and universities in order to build investigation, theory and practice of public health promotion (HP) and intervention.^2^

Given the necessity of HP stated in the 1986’s Ottawa’s Charter, public health policies and practices have been studied, formulated, tested and implemented in many nations.^3^

The world has been suffering from an epidemic of obesity and other chronic non-communicable diseases (CNCD) which are undoubtedly linked to adoption of unhealthy food, drinking, and behavioral lifestyles.^4-7^

In this regard, important findings on IR have been linked to face the obesity epidemic as well as the major risk factors for CNCD. Then, IR on obesity prevention should consider the following conceptions (Table 1).^8-10^

## Methods

In this narrative case report, the positive Brazilian experiences and pitfalls on IR regarding promotion of healthy lifestyles were updated and discussed.

Although no systematic review had been performed, studies were searched using three databases (MEDLINE, LILACS and Google Scholar) from 2006 to 2017 using the following terms: health promotion; Brazil; physical activity; dietary habits; sedentary behavior.

## Health Promotion Scenario

Considering the growing burden of CNCD and need of HP in Brazil, the National Police of Health Promotion (PNPS) was proposed in 2006.^11^

Nowadays, the PNPS comprises the following community integrated actions to foster^12^:

(a) healthy eating habits

(b) alcohol consumption reduction

(c) physical activity

(d) smoking reduction

(e) road safety

(f) peace and non-violence

(g) healthy environment

(h) vaginal delivery

The Brazilian PNPS have focussed on three strategic activities as follows:

- Health promoting schools
- Promotion of healthy dietary practices
- Promotion of physical activities and exercise

## Health promoting schools in Brazil

In 1995, the World Health Organization (WHO) launched the WHO Global School Health Initiative.^13^ A Health-Promoting School (HPS) is a special unit with improved capacity for being a healthy setting for living, learning and working.^14^ In 2007, WHO pointed the following elements for implementation of HP schools^15,16^:

- Building evidence and experience;
- Strengthening implementation processes in HPS;
- Alleviating social and economic disadvantage;
- Harnessing media influence on youth; and
- Improving partnerships among sectors/organizations.

At least 80 nations adopted and implemented the HPS initiative, especially in the Americas.^14,17-19^

Established in 2007, the Brazilian “Program Health at School” (“Programa Saúde na Escola”, PSE) was implemented only in 2012 and there are very few reports regarding its result and impact.^20^

A study realized in Recife, Northeastern Brazil, showed only 13.8% of implementation of HP activities such as healthy eating and physical activities in public schools, revealing many barriers for HP.^21^

In many Brazilian inner cities, the PSE was announced but not implemented. In this regard, populations from those cities have also excessive levels of unhealthy behaviors such as poor physical activity levels, insufficient sleep, smoking and alcohol drinking.^22^

## Promotion of healthy dietary practices in Brazil

Brazilian State has many different initiatives for encouragement of healthy dietary patterns included in the PNPS and the National Program of School Eating (PNAE) which was born in the 1940s.

Beyond the governmental actions, there are also very interesting community and school activities regarding farming and nutrition education. In many Brazilian communities foods and medicinal plants are cultivated in home and community gardens following sustainable models of the family agriculture and organic production.^23-27^ A long time ago this sustainable agriculture practices have been taught in Brazilian farming schools.^25,27^

Evaluating healthy eating in 173 public schools and 122 private schools in Federal District of Brazil, only one elementary private school matched 20 of 24 the established criteria for healthy eating among schools.^28^ In the same study, only 5 schools (1.7%) have healthy environments; just 4 schools (1.36%) reached the component “participation on school community”, and also in only 5 schools (1.7%) it was found a partnership with health sector and monitoring of nutritional status of schoolchildren.

## Promotion of physical activity: The successful Brazilian experiences

It has been suggested that sedentary behavior is declining in Brazil. But, the national prevalence of leisure-time physical activity is still only 15%.^29^

Notwithstanding, practice of physical exercise is one fundamental pillar of HP since regular engagement on physical activities improves cardiovascular system, prolongs life-span and decreases the risk of CNCD.^30^

Considering this scenario, a pioneering initiative was launched in the 1996 by the CELAFISCS.^31,32^ The *Agita São Paulo Program* (ASPP) is a successful example of IR outside of a developed country. During the 1999–2002 period, this physical activity program increased physical fitness 9.5% to 24% in the most populated Brazilian State– Sao Paulo.^31^ With a simple marketing message of daily engagement on 30-minutes of physical activities, exercises, and sports, especially walking and dancing, ASPP helped to decrease by 50% hospitalization by both hypertension and stroke, and by 57% type 2 diabetes mellitus in Sorocaba, a city of 600 000 inhabitants in São Paulo State.^33^ The ASPP has being implemented in many other countries around the world.^34^

In 2011, the Brazilian federal government put in practice a national program to confront obesity and CNCD. One of the strategic priority was the promotion of an active life through regular practice of leisure-time physical activity.^35,36^

Following this approach, the Program Academy of the City (AC) (or Academy of Health) is a community-based governmental intervention to for promotion of regular physical activities. It has been implemented in diverse Brazilian cities, but few reports are available. The reports covered the Brazilian state capitals Recife (PE), Curitiba (PR), Vitoria (ES), Aracaju (SE), Belo Horizonte (MG), and Rio Claro (SP), a country town.

In Recife, Northeastern Brazil, the AC was executed in 21 public areas (beaches, parks, recreation centers), and decisively contributed to increase physical activity among Recife’s population.^37^ However, the AC suffered from security problems related to urban violence, safety problems due to pitfalls in physical structure and necessity of maintenance and acquisition of equipment.^38^

In Aracaju, AC began in 2004 and reached 5000 people in five years among 15 city regions,^39^ whereas in Rio Claro, it successfully reduced the use of medicines, blood glucose, and blood pressure among physical activity practitioners.^40^

In Belo Horizonte, AC was implemented in 2005 and improved the dietary habits and physical activity levels of 400 people.^41^

In Curitiba, South Brazil, the municipal secretaries of “sports and leisure” and “Health” offered free of charge a great number of physical activities for communities by the “CuritibAtiva”, a sustainable and supportive leisure program which have been associated with an increased physical activity levels of that population.^42,43^

Another interesting Brazilian initiate is “walking for health” which covered three Brazilian capitals: Curitiba, Recife, and Vitoria. The percentages of attendants meeting the recommended daily physical activity needs were higher in Recife (16%), compared to Curitiba (9.6%), and Vitoria (8.8%). Regular practice of 150 minutes per week or more of walking was significantly associated with better self-rated health, younger age, and higher educational level,^44^ which means that less privileged people remain physically inactive due to work overload and lacking of leisure time as noted in capitals as well as in inner Brazilian cities.^41,45,46^

Considering the feasibility of promotion of physical activity in schools^47^ there is little programs in Brazil focusing this aspect. The “Saude na boa” program in ten schools from Florianopolis (SC) and Recife, increased physical activity levels of the adolescents.^48^

There are two specific programs for the elderly. The “Vamos program: active living, enhancing health”, and the “Third Age Academies”.^49,50^

The Vamos Program has been associated with improved prevalence of regular physical activity among middle-age and elderly Brazilians.^51^ Improving physical activity and diet among elderly people is an essential and cost-effective intervention for this target population.^4,52-54^

Among 116 countries, only 12% of nations, including Brazil and Iran, had explicit policies to decrease dietary fats and salt intake, to improve the intake of fruits and vegetables and promotion of regular physical activity.^55^

Another Brazilian successful health promotion initiative is The World Day of physical activity, created by CELAFISCS in Brazil as “Agita Mundo”. Today, this global initiative is done into the five continents among more than 70 countries.^56^

## Discussion and Critique

In spite of the governmental HP programs, Brazilian population has a raising dietary intake of fried foods, cholesterol-rich processed foods, sugars, soft drinks, confectionery, and fats, instead of adoption of a nutritional breakfast, and increased intake of fruits, vegetables, and a variety-rich diet.^57-63^

Following the example of many other developing and developed countries, Brazil has sponsored leisure-time physical activity and healthy eating programs^56,57^ which were not still capable of promoting massive participation, especially among workers, elderly, indigenous and less privileged socioeconomic classes.^38,45,64,65^

## Conclusion

Although successful exercise promotion strategies have been done in Brazil, the implementation science on promotion of healthy lifestyles is still rising and more research is necessary to identify the settings, circumstances, and processes by which sustainable adoption of healthy dietary and body practices occurs.^64^ Necessities and priorities regarding strengthening for implementation of healthy lifestyle are summarized in Table 2.

## Ethical approval

Not Applicable.

## Competing interests

There are no conflicts of interest.

## Funding

It was financed by the own author.

## Authors’ contributions

The author contributed alone from the conception and design of the study to the acquisition of data, writing and the final revision of the text.

**Table 1:** Emerging concepts on implementation research for obesity prevention in developing nations

Obesity police research is still rising
Urgency in taking policy preventive actions using mass-media strategies
Necessity of economic research on the theme
Need to investigate quasi-experiments and “natural experiments” from police-based interventions
Need of implementation research beyond the individual level of changing behavior
Improvement of public health educational level regarding behavior change and adoption of healthy lifestyles
Encouragement of individual and social empowerment since it improves adaptability, hope, desires, motivation, wellness, stress control, self-image and self-knowledge, know-how, and problem solving ability

Based on the studies of McKinnon et al^8^ and Taddeo Pda et al.^9^

**Table 2:** Priority settings for strengthening of lifestyle-related public health policies

**Promotion of healthy dietary practices**	
Weaknesses: Lack of nutrition education in public and private schools; Lack of teachers with basic knowledge regarding basic food and nutrition; Poor knowledge of children regarding healthy eating; Poor knowledge of families regarding healthy eating choices	Needs: Inclusion of dietitians as school teachers; Improvement of teachers' knowledge regarding healthy eating and obesity prevention; Implementation of a public and private task force to trigger better eating practices to decrease overweight and obesity in children and adults
**Health promoting schools**	
Weaknesses: Lack of implementation of "Saude na Escola" in many municipalities; Absence of program integration with educational sector; Lack of control regarding program implementation and financial costs; Lack of a complete health examination of students	Needs: Strengthening of integration with educational secretaries and community engagement; Necessity of promotion of healthy eating; Necessity of anthropometric and health evaluation; Evaluation of program coverage and impact; Inclusion of psychologists in multidisciplinary teams to foster behavior improvement of children and adolescents in order to adopt healthy lifestyles
**Promotion of leisure-time physical activities**	
Weaknesses: Lack of implementation of public programs on physical activity in all Brazilian Federative States; Insufficient structure for regular practice of free living physical activities; Lack of governmental advertising regarding benefits of physical activity	Needs: Increasing and improvement of public structure for practice of leisure-time physical activity; Integration of governmental initiatives with regional and local settings, including schools and community centers; Public and private investment on public structure for practice of sports and exercises
